# A Systematic Review of the Use of Artificial Intelligence Systems for Assessing Multiple Parameters During the Ultrasound Evaluation of Fetal Growth

**DOI:** 10.3390/diagnostics16183063

**Published:** 2026-09-21

**Authors:** Oana Cârlig, Cătălina Iovoaica-Rămescu, Florin Claustel Jurma, Dominic Gabriel Iliescu

**Affiliations:** 1 Doctoral School, University of Medicine and Pharmacy of Craiova, 20039 Craiova, Romania; 2 Department of Obstetrics and Gynecology, University of Medicine and Pharmacy of Craiova, 200349 Craiova, Romania; 3 Facultatea de Informatică, Universitatea de Vest Timișoara, 300223 Timișoara, Romania; 4 Department of Obstetrics and Gynecology, Emergency County Hospital of Craiova, 200642 Craiova, Romania

**Keywords:** artificial intelligence, fetal biometry, obstetric ultrasonography, automated fetal biometry, deep learning, fetal growth

## Abstract

**Background/Objectives**: Artificial intelligence (AI)-assisted automatic fetal measurements during ultrasound evaluation have become a major subject of interest. Most publications include systems developed and tested for a single anatomical component. In routine clinical practice, multiple biometric parameters are required for fetal weight estimation. Our objective is to assess the studies that incorporated three or more standard plane fetal automatic measurements with the help of AI systems in the second and third trimesters. **Methods**: We performed a systematic review of the published research focusing on automated AI systems analyzing at least three standard fetal biometric parameters or their corresponding standard ultrasound planes for fetal biometric, gestational-age, or fetal-growth assessment. The literature screening had to meet the predefined inclusion and exclusion criteria. **Results**: Nineteen studies, comprising more than 730,000 ultrasound images, met the inclusion criteria, and eighteen studies compared automated measurements with manual reference measurements. Overall, the included studies reported favourable performance for automated assessment of biparietal diameter, head circumference, abdominal circumference, and femur length, although substantial heterogeneity was observed in study design, AI architecture, validation methodology, and reported performance metrics. **Conclusions**: Automated AI systems show promising performance for assessing multiple standard fetal biometric parameters and may improve measurement consistency and examination efficiency. However, substantial heterogeneity in datasets, model architectures, and outcome reporting restricts direct comparison and the certainty of the evidence. Standardized reporting and prospective multicentre evaluation across gestational ages (GA), ultrasound systems, and operators are needed before these systems can be recommended for routine clinical use.

## 1. Introduction

The term ‘artificial intelligence’ (AI) has become a part of our common language and is increasingly used alongside medical terminology. AI consists of all computer processes used to accomplish tasks that would normally require human intelligence. It extends into numerous fields of interest such as computer vision, natural language processing, speech and audio processing, autonomous vehicles and robotics, medical imaging and diagnostics, and many other scientific and even humane applications.

To make this possible, deep learning models rely on multiple interconnected computational layers that resemble neural networks [1]. This is a part of a type of machine learning called a neural network that is best used for identifying complex patterns or connections within large datasets. Convolutional neural networks (CNNs) are supervised learning systems that make their predictions through learned mapping [2].

Regarding medical image analysis, supervised techniques are being developed to help process images [3] for segmentation [4], detection [5], and reconstruction [6], as well as various other applications [7]. This helps researchers to dive into a complex set of actions that include assigning labels to pixels, generating bounding boxes, and creating a visual representation from signals acquired by medical devices [3].

Based on recent studies, AI has been developed to aid obstetric ultrasound for a better estimation of gestational age, standard plane acquisition, fetal biometry, placenta and amniotic fluid assessment, and detection of fetal anomalies.

Despite more than six decades of clinical obstetric ultrasonography [8], the optimal approach to ultrasound-based fetal weight estimation remains a subject of ongoing investigation. This is primarily due to the methodological heterogeneity among the studies focusing on fetal biometry [9]. Another important factor is the intra- and interobserver variability [10] regardless of the worldwide consensus regarding the standard planes needed for biometry measurements [11,12].

The International Society of Ultrasound in Obstetrics and Gynecology (ISUOG) guideline identifies biparietal diameter (BPD), head circumference (HC), abdominal circumference (AC), and femur length (FL) as the standard biometric measurements used to calculate the estimated fetal weight (EFW). The guideline makes recommendations in regard to the collected data, and it includes all the four biometric measurements used in the Hadlock formula [11]. Nevertheless, there are four variations of this formula with differences regarding the accuracy of the estimated fetal weight. In order to have the most appropriate approach, we will take into consideration the ISUOG guideline recommendations [11] and the INTERGROWH-21st standards [13,14].

One of the main research interests in recent years is to create systems that would automatically detect and measure the biometric parameters during fetal ultrasound and thus reduce both the intra- and interobserver variability and the examination time. Our aim is to conduct a comprehensive systematic review of the studies focused on the use of artificial intelligence systems for the automatic assessment of multiple standard plane fetal biometric parameters on the antenatal ultrasound.

## 2. Materials and Methods

### 2.1. Study Setting and Design 

This systematic review was conducted in accordance with the Preferred Reporting Items for Systematic Reviews and Meta-Analyses (PRISMA 2020) guidelines [15]. It synthesizes the studies focusing on AI use for the automatic detection and measurement of the standard biometric parameters that are noted on the obstetric ultrasound from the second trimester onward: head circumference (HC) and/or biparietal diameter (BPD), abdominal circumference (AC), and femur length (FL). Until further research concludes otherwise, we will use the sonographers’ manual measurements as the reference standard for comparison.

### 2.2. Search Methods and Information Sources

A systematic literature search was conducted to identify studies evaluating artificial intelligence-based automated analysis of multiple standard fetal biometric parameters or their corresponding ultrasound planes. The search strategy combined controlled vocabulary and free-text terms relating to four principal concepts: the fetus or pregnancy; prenatal ultrasonography; artificial intelligence, machine learning or automated image analysis; and fetal biometry, biometric measurement, gestational-age estimation or fetal-weight assessment. The search strategy included the following combination of terms: ‘fetal’ or ‘obstetric’ or ‘prenatal’ or ‘antenatal’; ‘ultrasound’ or ‘ultrasonography’ or ‘sonography’; ‘artificial intelligence’ or ‘neural networks’ or ‘deep learning’ or ‘convolutional networks’ or ‘neural networks’ or ‘machine learning’ and/or ‘automatic detection’ or ‘automatic measurement’; and ‘biometry’ or ‘biometric parameters’. The electronic search was designed to maximize sensitivity; therefore, the requirement for analysis of at least three standard fetal biometric parameters or corresponding standard planes was applied during study screening rather than incorporated as a search restriction. PubMed and Scopus were queried for studies published between inception and 25 February 2026. Google Scholar was used as a supplementary source to identify potentially eligible records not retrieved from the primary bibliographic databases. Studies published in English were eligible for inclusion. Source-specific retrieval counts were not recorded separately; therefore, the combined number of records identified is reported. Before conducting the final searches, we tested each strategy to determine whether it retrieved a set of eligible studies already known to the reviewers. When a known eligible study was not retrieved, the search terms were revised to improve sensitivity. Study titles and authors’ names were not included as search terms. The studies that met the inclusion criteria were selected. Each study then underwent assessment and was categorized as relevant, possibly relevant, or not relevant. The possibly relevant studies were assessed for eligibility, and only pertinent publications were incorporated into our study. Any discrepancies were deliberated and resolved by consensus between two researchers. Specific exclusion criteria were applied to identify the most pertinent studies. Complete database-specific search strategies for PubMed and Scopus are provided in Supplementary Materials. To improve reproducibility, these strategies were reconstructed from the original search concepts and predefined eligibility criteria and rerun during manuscript revision.

### 2.3. Eligibility Criteria

#### 2.3.1. Inclusion Criteria

Studies were eligible if they evaluated fully automated AI systems analyzing at least three standard fetal biometric parameters or their corresponding standard ultrasound planes (biparietal diameter, head circumference, abdominal circumference, and femur length) for fetal biometric, gestational-age, or fetal-growth assessment. Only studies published in the English language were considered eligible for inclusion. We used no restrictions regarding women’s age or race, or the type of ultrasound machine. We included all studies that evaluated three or more of the ultrasound measurements used for fetal growth, regardless of the preferred fetal growth formula.

The eligibility criteria and review methodology were defined before study screening; however, a formal protocol was not registered in a systematic review registry.

#### 2.3.2. Exclusion Criteria

Studies focusing exclusively on first-trimester ultrasound, fetal anomaly detection, placental assessment, or amniotic fluid evaluation, as well as review articles, were excluded. Studies concerned solely with the acquisition or detection of standard fetal ultrasound planes were also considered outside the scope of this review. As the review focused on AI systems capable of assessing multiple standard fetal biometric parameters, studies limited to a single biometric parameter or to non-standard fetal biometric parameters were excluded. Semi-automated approaches requiring manual intervention in the computational analysis or measurement process, including manual region-of-interest initialization, landmark placement, contour correction, or calliper placement, were also excluded.

### 2.4. Data Collection

Study eligibility was assessed independently by two reviewers through screening of titles, abstracts, and, subsequently, full-text articles. Differences in study inclusion were discussed between the reviewers and resolved by consensus. Data extraction—including details regarding biometric parameters, study design, network architecture, and reported performance outcomes—was performed by one author and independently verified by a second author. No randomized controlled trials met the inclusion criteria, as all eligible studies were observational or algorithm development designs.

The primary outcome of interest was the performance of automated AI systems analyzing at least three standard fetal biometric parameters or their corresponding standard ultrasound planes for fetal biometric, gestational-age, or fetal-growth assessment. Eligibility was based on the complete methodological description provided in the full-text article rather than solely on the primary model output reported in the abstract. The biometric parameters of interest were biparietal diameter (BPD), head circumference (HC), abdominal circumference (AC), and femur length (FL). Measurement performance was extracted using the metrics reported by the original studies, including absolute error, mean absolute error (MAE), mean absolute percentage error (MAPE), mean absolute relative error (MARE), and other measures of agreement or measurement discrepancy. Where reported, segmentation performance, including the Dice similarity coefficient (DSC) and Jaccard index, was collected as a secondary outcome. All eligible results reported for the biometric parameters of interest were extracted; where metrics were not directly comparable, results were retained in their originally reported form rather than converted or combined.

In addition to outcome data, the following study characteristics were extracted: author and year of publication; fetal biometric parameters assessed; study population or dataset size; AI model or network architecture; methods used for anatomical detection and segmentation; use of a manual reference standard, scale-retrieval method, image resizing or preprocessing; ultrasound equipment; and gestational-age range, where available. Information not reported by the original authors was recorded as not reported, and no assumptions or imputation were made for missing or unclear study characteristics.

Reporting quality and completeness were assessed by one reviewer using the Checklist for Artificial Intelligence in Medical Imaging (CLAIM 2024). Each applicable CLAIM item was rated as Yes, No, or Not Applicable according to the checklist guidance. Because CLAIM is a reporting guideline rather than a dedicated risk-of-bias instrument, no aggregate CLAIM score or risk-of-bias classification was derived from the checklist. For studies developing and/or evaluating clinical prediction models, methodological quality, risk of bias, and applicability were additionally assessed by two reviewers using PROBAST+AI (Prediction model Risk Of Bias Assessment Tool). In accordance with PROBAST+AI, model development was assessed for methodological quality and applicability, whereas model evaluation was assessed for risk of bias and applicability. Studies were classified as reporting model development, model evaluation, or both, and the corresponding PROBAST+AI components were applied. PROBAST+AI was not applied to studies whose AI systems were designed primarily for anatomical detection, segmentation, automated calliper placement, or biometric measurement without development or evaluation of a clinical prediction model, because these designs fall outside the intended scope of the instrument. Given the heterogeneity of these technical AI studies and the absence of a single validated risk-of-bias instrument applicable across all such designs, no alternative risk-of-bias tool was imposed on these studies.

### 2.5. Eligibility for Synthesis

Studies were grouped for synthesis according to the fetal biometric parameters evaluated (BPD, HC, AC, and FL) and the reported performance outcomes. Studies contributed only to analyses for which the corresponding biometric parameter and performance metric were available. Additional methodological characteristics, including automatic detection, segmentation, and scale retrieval, were synthesized separately.

Reported performance measures were extracted using the definitions provided by the original authors. Percentage-based errors and gestational-age prediction errors were retained in their original form and were not converted into absolute biometric errors. Segmentation metrics, including Dice and Jaccard coefficients, were analyzed separately from biometric measurement errors. No imputation of missing outcome data was performed.

Study characteristics and outcomes were summarized in structured tables. Studies were tabulated according to author and year, biometric parameters evaluated, sample size, AI architecture, degree of automation, image preprocessing, and reported performance metrics. Measurement-error outcomes and segmentation-performance metrics were presented separately to facilitate comparison across studies.

Owing to substantial methodological and clinical heterogeneity across the included studies, a statistical meta-analysis was not considered appropriate. Studies differed considerably in AI architecture, dataset size, image acquisition, reference standards, and reported performance metrics. Consequently, results were synthesized narratively and descriptively, with measurement errors summarized separately for BPD, HC, AC, and FL. Where multiple studies reported comparable metrics, the range of reported values was presented without calculation of a pooled effect estimate.

Potential sources of heterogeneity were explored descriptively by comparing study characteristics, including dataset size, ultrasound equipment, AI architecture, biometric parameters assessed, and degree of automation. Formal subgroup analyses or meta-regression were not performed because of the limited number of studies and substantial heterogeneity in reported outcomes and performance metrics. No formal sensitivity analyses were performed because no quantitative meta-analysis was undertaken.

## 3. Results

**Figure 1:**
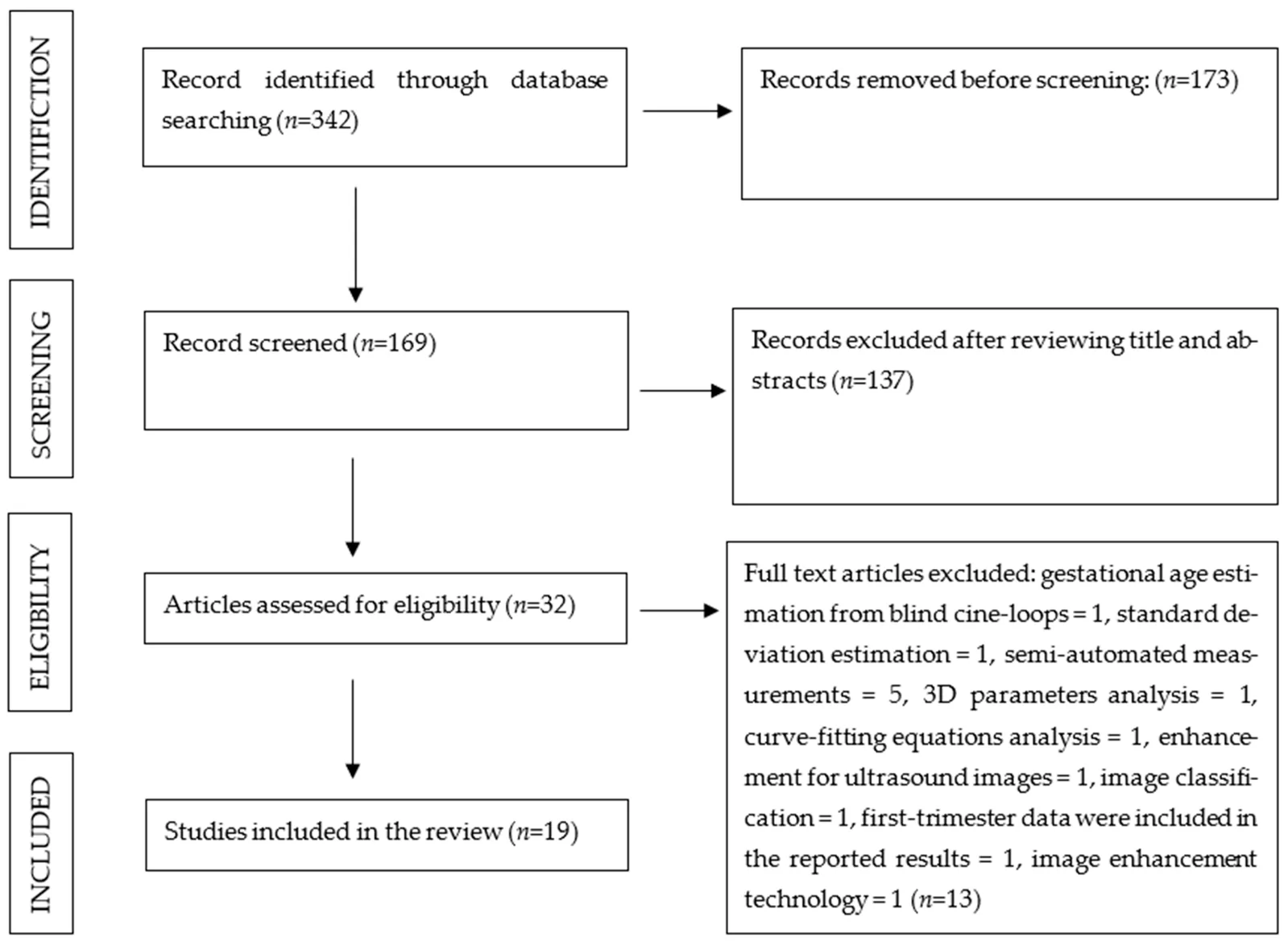
PRISMA flow diagram of the study selection process detailing the number of database findings, exclusion criteria, and articles included in the review.

From the preliminary database findings, 173 records were disregarded due to the main exclusion criteria (records marked as ineligible by automation tools, records removed for other reasons). The remaining 169 records had their titles and abstracts screened, and we eliminated the ones focused solely on the assessment of fewer than three biometric parameters, first-trimester ultrasound, fetal anomaly detection, placental assessment, amniotic fluid evaluation, standard plane detection, and anatomical structure analysis. Thirty-two records underwent full-text review. Thirteen were subsequently excluded because their primary focus was gestational age estimation from blind cine-loops [16], standard deviation estimation [17], semi-automated measurements [18,19,20,21,22], 3D parameter analysis [23], curve-fitting equation analysis [24], enhancement for ultrasound images [25], image classification [26], first-trimester data that were included in the reported results [27], or image enhancement technology [28]. At the end of the selection process, 19 records were identified as being fit for complete inclusion in the analysis. We synthesised the selection process following the Preferred Reporting Items for Systematic Reviews and Meta-Analyses (PRISMA) guidelines in Figure 1.

All 19 included studies were assessed using CLAIM 2024. Reporting completeness varied across studies, with the most frequently unmet items relating to intended sample size, testing on external data, handling of missing data, measures of inter- and intra-rater variability of features described by the annotators, software libraries, frameworks, and packages. Studies meeting the definition of clinical prediction-model studies were additionally assessed using PROBAST+AI. For these studies, methodological quality of model development and risk of bias in model evaluation were considered separately, together with concerns regarding applicability. PROBAST+AI was considered not applicable to the remaining studies, which primarily evaluated automated detection, segmentation, or biometric measurement algorithms.

The majority of the studies reported a total of 734.367 ultrasound images, with a small subset providing case numbers alone [29,30]. The four-parameter evaluation was reported in 13 articles, and three-parameter assessment was noted in six studies in which the abdominal circumference and femur length were consistently evaluated, and only one parameter was reported for the fetal head (head circumference or biparietal diameter). We did not make any notes regarding any growth formula used, since the focus was on the performance of AI systems in automatically detecting and measuring multiple parameters within the ultrasound examination. All authors described the network architecture used, with the earlier authors providing an elaborate description that had fewer denominations for the segmentation models and the latest publications mentioning well-established networks with less description of the models. The biometric parameters assessed and the network architecture used are summarized in Table 1.

Although two studies did not explicitly describe the automatic detection or segmentation steps, both of which are integral to fully automated biometric assessment, all included studies reported the use of automated fetal measurements. Comparison with manual measurements was noted in 18 articles, with one author performing a comparison with other network architectures. The details for automatic detection, segmentation, measurements, and scale retrieval are summarized in Table 2.

Four studies did not describe any scale retrieval or image resizing, with three of them expressing results in millimetres. This suggests that image acquisition relied on the manufacturer’s default ultrasound configuration. The one study that did not express results in the metric system was the first article published regarding an automatic system used for multi-parameter evaluation (Fernández-Caballero, A. Lecture Notes in Computer Science, vol 2085. 2001), with results seen as highly experimental [29].

### 3.1. Analysis of Primary Outcomes

The primary outcome was the agreement between automated and reference fetal biometric measurements. Where available, measurement performance was extracted as absolute error, mean absolute error (MAE), mean relative error (MRE), mean absolute percentage error (MAPE), mean squared deviation (MSD), or mean absolute relative error (MARE). For studies reporting segmentation performance, Dice similarity coefficient (DSC), Jaccard index, and related overlap measures were extracted as secondary performance measures. Results were retained in their originally reported metrics where conversion to a common measure was not methodologically appropriate.

Across the 18 studies reporting comparisons with reference measurements, automated fetal biometry generally demonstrated small measurement discrepancies (MAE 0.17–3.3 mm for BPD, 1.08–10.4 mm for HC, 0.49–12.0 mm for AC, and 0.03–3.14 mm for FL), although substantial heterogeneity in study design, performance metrics, and validation methods precluded statistical pooling. Reported performance metrics were harmonized by grouping them into agreement indices (accuracy, Williams index), Jaccard index, disagreement indices (absolute error, mean absolute error, mean absolute percentage error, and mean absolute relative error), and Dice similarity coefficient. Dice similarity coefficient (DSC) is a formula used to gauge the similarity between two samples. Seven studies calculated the DSC, with values varying from 0.95 to 0.98 for fetal head, 0.90 to 0.98 for abdomen and slightly worse outcomes for FL, with values as low as 0.73. The detailed results are outlined in Table 3.

As shown in Table 3, considerable variability was observed across studies in terms of the evaluated biometric parameters, AI approaches, and reported performance measures. Despite these methodological differences, the included studies generally demonstrated the feasibility of AI-assisted fetal biometric assessment. However, direct comparison between studies remains limited by differences in study design, datasets, validation approaches, and outcome reporting.

### 3.2. Sources for Heterogeneity

Potential sources of heterogeneity were explored descriptively by comparing study characteristics, including gestational-age range, dataset size, ultrasound equipment, AI architecture, biometric parameters assessed, degree of automation, and validation methodology. Formal subgroup analyses or meta-regression were not performed because of the limited number of studies and substantial heterogeneity in reported outcomes and performance metrics.

Eleven authors mentioned the ultrasound machines used for the fetal image collection. None of the studies offered validation tests with ultrasound machines other than the ones used for primary testing.

Because the included studies were too methodologically and clinically diverse for meta-analysis, we did not perform a formal sensitivity analysis. Instead, we considered the robustness of our narrative conclusions by examining the findings across studies with different designs and methods.

Results analysis for each publication included agreement indices, disagreement indices, and/or Dice similarity coefficient. We can note the substantial heterogeneity in reported outcomes and performance metrics.

## 4. Discussion

This systematic review identified 19 studies evaluating AI systems for the automated assessment of multiple standard fetal biometric parameters during second- and third-trimester ultrasound examinations. The focus on multiple parameters reflects routine obstetric practice, where several standard biometric measurements are used for fetal weight estimation. Overall, the available evidence suggests that automated systems can achieve relatively small measurement discrepancies compared with reference measurements. However, substantial heterogeneity in study populations, AI architectures, validation methods, and reported outcomes limits direct comparison between studies, while evidence regarding external validation across different ultrasound systems, operators, and clinical settings remains limited.

AI systems for automated fetal biometry have progressed considerably over the past two decades, from early experimental approaches to more recent systems based on deep learning architectures and automated clinical workflows. The included studies used a variety of approaches, including convolutional neural networks, U-Net-based architectures, multi-task learning and attention mechanisms, and generally reported favourable performance in the automated assessment of standard fetal biometric parameters. Nevertheless, differences in study design and validation methodology make direct comparisons of model performance difficult.

Across the included studies, the lowest measurement discrepancies were generally observed for biparietal diameter (BPD) and femur length (FL), whereas greater variability was observed for head circumference (HC) and abdominal circumference (AC). One possible explanation is the greater complexity of contour-based measurements. In contrast to BPD, which is derived from two defined calliper points, and FL, which is predominantly a linear measurement, circumference estimation requires delineation of a larger anatomical boundary. Although this difference in measurement approach may be relevant when interpreting the observed results, the included studies did not systematically investigate the causes of parameter-specific differences in automated measurement performance. Therefore, no definitive explanation for the observed variation can be established from the available evidence. Measurement performance may also vary across gestational age, emphasizing the importance of evaluating automated systems across the full gestational-age range for which they are intended [36].

Although the reported measurement errors were generally small, their clinical relevance cannot be determined from numerical error alone. Differences in individual biometric measurements may influence derived parameters such as estimated fetal weight and gestational-age assessment. Therefore, future validation studies should assess not only agreement with reference measurements, but also whether the observed measurement differences are sufficiently small to preserve the clinical interpretation of fetal biometry.

The substantial variation in dataset size should also be considered when interpreting the reported performance. Although recent studies have benefited from substantially larger datasets [32], a large number of ultrasound images does not necessarily represent equivalent clinical diversity, as multiple images may originate from the same examination or patient. The number and diversity of independent patients, centres, operators and ultrasound systems represented in the validation dataset may therefore be more informative for assessing generalizability than image count alone.

Generalizability across ultrasound equipment represents another important limitation of the available evidence. Eleven studies reported the ultrasound systems used for image acquisition [30,35,37,38,40,41,42,43,44,45,46], but none reported external validation using ultrasound systems different from those used for primary testing. Differences in image acquisition, processing, and manufacturer-specific image characteristics may affect the performance of image-analysis algorithms. Consequently, a model that performs well on the ultrasound systems used during development may not achieve the same performance when applied to different ultrasound platforms.

The heterogeneity of reported performance metrics represents an additional barrier to comparison between AI systems. Measurement error, percentage error, segmentation overlap metrics, and gestational-age prediction error describe different aspects of model performance and should not be interpreted interchangeably. In particular, Dice and Jaccard coefficients quantify spatial overlap between segmentations but do not by themselves establish the clinical acceptability of the resulting biometric measurements. Future studies would benefit from reporting standardized measurement-error outcomes alongside segmentation metrics and clinically relevant measures of agreement. The substantial differences in reported outcomes and validation methods observed in this review also prevented meaningful quantitative pooling and therefore supported the use of narrative rather than meta-analytic synthesis.

The degree to which individual computational steps were reported also varied considerably between studies. Although many automated biometry systems incorporate anatomical detection followed by segmentation or landmark localization before deriving a biometric measurement, these intermediate steps are not necessarily explicit in all model architectures. Several included studies provided insufficient information regarding detection or segmentation [30,32,42]. Clear reporting of the complete computational pipeline is important for reproducibility and for understanding potential sources of measurement error, particularly when comparing systems based on different AI architectures.

The reporting-quality assessment further highlighted limitations in the available evidence. Using CLAIM 2024, commonly underreported elements included intended sample size, external testing, handling of missing data, assessment of inter- and intra-rater variability, and details of the software libraries, frameworks and packages used. These reporting gaps make it more difficult to assess the reproducibility and generalizability of the reported systems. More consistent adherence to AI-specific reporting guidance, together with clearer descriptions of datasets, validation procedures and performance metrics, would improve transparency and facilitate comparison and independent validation of automated fetal biometry systems. It is important to emphasize that CLAIM is a reporting guideline designed to assess the completeness and transparency of reporting in medical imaging AI studies and is not a risk-of-bias assessment instrument.

## 5. Future Directions

Although the available evidence demonstrates excellent performance of AI-assisted fetal biometry, several challenges remain before widespread clinical implementation can be achieved. Future research should prioritize prospective multicentre validation studies, external validation across different ultrasound systems and operators, and the adoption of standardized reporting of datasets, validation methodologies, and performance metrics. Such efforts would improve comparability between studies, facilitate independent validation, and accelerate the safe integration of AI-assisted fetal biometry into routine obstetric ultrasound practice. Ultimately, the goal of artificial intelligence is not to replace the sonographer, but to improve measurement consistency, reduce examination time, and support clinical decision-making.

## 6. Limitations

We acknowledge the absence of prospective registration as a methodological limitation. However, the review methods, eligibility criteria, study-selection process, and quality appraisal procedures are reported transparently in accordance with PRISMA 2020.

PROBAST+AI was assessed in only four studies due to substantial methodological heterogeneity and the absence of a universally accepted appraisal tool applicable across all study designs. Considerable heterogeneity in datasets, ultrasound equipment, and performance metrics limited direct quantitative comparison. Most included studies were retrospective.

## Figures and Tables

**Table 1:** General characteristics of the studies included in the systematic review. The Table summarizes the included studies according to publication year, fetal biometric parameters evaluated, study population or dataset size, and the artificial intelligence architecture used for automated fetal biometry.

Author, Year	Biometric Parameters Assessed	Number of Patients/Images	Network Architecture
Goetz-Fu, M. 2025 [31]	HC, AC, FL	6299 images	EfficientNetB2 (a U-Net++ architecture)
Mikołaj, K.W. 2025 [32]	BPD, HC, AC, FL	433.096 images	RegNetX 400 Mf-based CNN
Parga, C.D.2025 [33]	BPD, HC, AC, FL	3293 images	AGE-US (a single-encoder double-decoder U-Net architecture)
Sundaramoorthy, K. 2025 [34]	HC, AC, FL	1437 images	Nested U-Net (U-Net ++) for HC training, Hybrid Faster R-CNN + HRNet + Attention U-Net for AC training, U-Net for FL
Venturini, L. 2025 [35]	BPD, HC, AC, FL & other	1457 images	A U-Net architecture
Degala, S.K.B. 2024 [36]	BPD, HC, AC, FL	3996 images	Attention Gate Double U-Net with Guided Decoder (ADU-GD)
Han, X. 2024 [37]	BPD, HC, AC, FL & other	642 images	CUPID (a traditional CNN architecture)
Cho, H. 2024 [38]	BPD, HC, AC, FL	6000 images	H-BiSeNet
Qazi, M.A. 2023 [39]	HC, AC, FL	5872 images	An end-to-end CNN
Slimani, S. 2023 [40]	BPD, HC, AC, FL	172.293 images	Mask-R-CNN (models: R-101-C4-3x, R-101-DC5-3x, R-50-C4-3X, R-50-DC5-3X)
Dan, T. 2023 [41]	BPD, HC, AC, FL	8378 images	DeepGA: DeepSeg (PointRend R-CNN) and DeepReg (uses AlexNet)
Lee, C. 2023 [42]	HC, AC, FL & other	7249 images	EfficientNet-B7
Płotka, S. 2022 [43]	BPD, HC, AC, FL	76.874 images	FUVAI (an encoder–decoder U-Net)
Oghli, M.G. 2021 [44]	BPD, HC, AC, FL	1807 images	Attention MFP-Unet
Bano, S. 2021 [45]	BPD, HC, AC, FL & other	349 images	AutoFB: U-Net and Deeplabv3+ (encoder–decoder CNN)
Salim, I. 2019 [46]	HC, AC, FL	891 images	The system developed by Philips research
Yazdi, B. 2014 [30]	BPD, AC, FL & other	95 pregnancies	SonoBiometry developed by GE Healthcare
Carneiro, G. 2008 [47]	BPD, HC, AC, FL & other	4434 images	A constrained probabilistic boosting tree
Fernández-Caballero, A. 2001 [29]	BPD, AC, FL	1 pregnancy	A lateral interaction in accumulative computation model

**Table 2:** Technical characteristics of the included artificial intelligence systems used for automated fetal biometry. The Table summarizes whether each study reported automatic fetal structure detection, automatic segmentation, automatic biometric measurement, comparison with manual reference measurements, scale retrieval, and image resizing or preprocessing before analysis.

Author, Year	Automatic Detection	Automatic Segmentation	Automatic Measure	Comparison to Manual	Scale Retrieval	Image Resize (Pixels)
Goetz-Fu, M. 2025 [31]	Yes	Yes	Yes	Yes	Yes	448 × 448
Mikołaj, K.W. 2025 [32]	No mention	No mention	Yes	Yes	Yes	224 × 224
Parga, C.D.2025 [33]	Yes	Partial. Not for FL	Yes	No	Yes	256 × 256
Sundaramoorthy, K. 2025 [34]	Yes	Yes	Yes	Yes	No	No
Venturini, L. 2025 [35]	No mention	Yes	Yes	Yes	Yes	384 × 288
Degala, S.K.B. 2024 [36]	Yes	Yes	Yes	With other networks	Yes	224 × 224
Han, X. 2024 [37]	Yes	Yes	Yes	Yes	No	no mention
Cho, H. 2024 [38]	Yes	Yes	Yes	Yes	Yes	512 × 512
Qazi, M.A. 2023 [39]	Yes	Yes	Yes	Yes	Yes	256 × 256
Slimani, S. 2023 [40]	Yes	Yes	Yes	Yes	Yes	shorter edge 800
Dan, T. 2023 [41]	Yes	Yes	Yes	Yes	Yes	227 × 227
Lee, C. 2023 [42]	No mention	No mention	Yes	Yes	Yes	320 × 240
Płotka, S. 2022 [43]	Yes	Yes	Yes	Yes	Yes	224 × 224
Oghli, M.G. 2021 [44]	Yes	Yes	Yes	Yes	No	800 × 540
Bano, S. 2021 [45]	Yes	Yes	Yes	Yes	Yes	512 × 512
Salim, I. 2019 [46]	Yes	Yes	Yes	Yes	Yes	no
Yazdi, B. 2014 [30]	Yes	No mention	Yes	Yes	No	no
Carneiro, G. 2008 [47]	Yes	Yes	Yes	Yes	Yes	78 × 78 and 78 × 30
Fernández-Caballero, A. 2001 [29]	Yes	Yes	Yes	Yes	No	No

**Table 3:** Results analysis for each publication, including agreement indices, disagreement indices and/or Dice similarity coefficient. We can note the substantial heterogeneity in reported outcomes and performance metrics.

Author, Year	Disagreement Indices (Absolute Error, MAE, MRE, MAPE, MSD, and MARE)	Other Metrics Included: Agreement Indices (Accuracy, Williams Index), Jaccard Index, and Dice Similarity Coefficient
M.Goetz-Fu, 2025 [31]	MARE 0.96% for HC, 1.56% for AC, 1.77% for FL	
Mikołaj, K.W. 2025 [32]	MRE 6.49% for appropriate gestational age	
Parga, C.D.2025 [33]	MAE 0.97 mm for BPD, 6.58 mm for HC, 12.0 mm for AC, 3.14 mm for FL MAPE 2.1% HC, 1.1% BPD, 3.8% AC, 5.4% FL Hausdorff distance	Dice index median 0.97
Sundaramoorthy, K. 2025 [34]	MAE 9.7 mm for HC, 5.4 mm for AC	Accuracy 0.89 for HC, 0.85 for AC
Venturini, L. 2025 [35]	MSD 2.60 mm for HC, 0.79 mm for BPD, 4.61 mm for AC, 0.97 mm for FL	
Degala, S.K.B. 2024 [36]	MAE in II^nd^ trimester 2.2 ± 1.9 mm for HC, 1.3 ± 0.6 mm for BPD, 1.8 ± 1.1 mm for AC, 1.2 ± 0.8 mm for FL MAE in III^rd^ trimester 2.0 ± 1.7 mm for HC, 1.1 ± 0.9 mm for BPD, 2.2 ± 1.8 mm for AC, 0.5 ± 1.1 mm for FL	Dice index median 0.99
Han, X. 2024 [37]	MAE 0.50 mm for BPD, 2.53 mm for HC. 2.22 mm for AC, 0.81 mm for FL Percentage difference 0.94% for BPD, 1.28% for HC, 1.25% for AC, 1.99% for FL	
Cho, H. 2024 [38]	MSD 5.37 for head, 8.71 for abdomen, 4.26 for FL	Accuracy 0.98 head, 0.99 abdomen, 0.99 femur Jaccard index 0.95 head, 0.92 abdomen, 0.77 femur Dice index 0.97 head, 0.95 abdomen, 0.86 femur
Qazi, M.A. 2023 [39]	MAE 1.08 mm for head, 1.44 mm for abdomen, 1.10 mm for femur	
Slimani, S. 2023 [40]	MAE 6.7 mm for HC, 3.3 mm for BPD, 9.1 mm for AC, 2.7 mm for FL	Dice index 0.95 head, 0.90 abdomen, 0.73 femur
Dan, T. 2023 [41]	MAE 0.77 weeks = 5.39 days	Dice index 0.98 head, 0.97 abdomen, 0.86 femur
Lee, C. 2023 [42]	MAE 3.97 days	
Płotka, S. 2022 [43]	MAE 2.7 mm for BPD, 10.4 mm for HC, 10.6 mm for AC, 2.0 mm for FL	Dice index median 0.96
Oghli, M.G. 2021 [44]		Accuracy 0.95 abdomen, 0.96 head, 0.94 femur Dice index 0.97 head, 0.98 abdomen, 0.91 femur
Bano, S. 2021 [45]	Absolute error 0.80 mm for BPD, 2,67 for HC, 3.77 mm for AC, 2.1 mm for FL	
Salim, I. 2019 [46]	MAE 2.1 mm for HC, 7.2 mm for AC, 2.1 mm for FL	88.9% automatic calliper placement appropriate + minor adjustments
Yazdi, B. 2014 [30]	MAE 0.17 mm for BPD, 0.49 mm for AC, 0.03 mm for FL	89.5% appropriate biometric measurements for BPD, AC, FL
Carneiro, G. 2008 [47]	Absolute error 1.11 mm for BPD, 5.07 mm for HC, 10.67 mm for AC, 0.89 mm for FL Percentage difference 1.46% for BPD, 1.25% for HC, 3.00% for AC, 2.11% for FL	Williams index BPD 0.82, HC 1.05, AC 0.70, FL 0.92
Fernández-Caballero, A. 2001 [29]	1 case, no number of images. Computed age BPD 17.6 weeks, FL 18.2 weeks	

## Data Availability

No new datasets were generated or analysed during the current study. All data included in this systematic review were obtained from previously published studies and are available from the corresponding cited sources. Data extracted for the review are presented within the article and its Supplementary Materials.

## Supplementary Materials

The following supporting information can be downloaded at: https://www.mdpi.com/article/10.3390/diagnostics16183063/s1.
