# Supplementary material for: A Systematic Review of the Use of Artificial Intelligence Systems for Assessing Multiple Parameters During the Ultrasound Evaluation of Fetal Growth
Source: Diagnostics (Basel). 2026 Sep 21;16(18):3063. doi: 10.3390/diagnostics16183063 (PMC13605745; doi:10.3390/diagnostics16183063)
Supplement: Supplementary file 1 [file diagnostics-16-03063-s001.zip › CLAIM percentages all authors.pdf]

CLAIM percentage

Author and year	CLAIM percentage
Venturini et al., 2025	90.0%
Sundaramoorthy et al., 2025	67.5%
Díaz-Parga et al., 2025	67.5%
Goetz-Fu et al., 2025	82.5%
Mikołaj et al., 2025	76.2%
Cho et al., 2024	60.0%
Degala et al., 2024	55.0%
Han et al., 2024	65.0%
Dan et al., 2023	82.9%
Lee et al., 2023	82.9%
Qazi et al., 2023	47.5%
Slimani et al., 2023	92.9%
Płotka et al., 2022	72.5%
Bano et al., 2021	55.0%
Oghli et al., 2021	62.5%
Salim et al., 2019	74.3%
Yazdi et al., 2014	55.9%
Carneiro et al., 2008	52.5%
Fernández-Caballero et al., 2001	30.0%

Percentage = Y / (Y + N) × 100. NA items excluded
