# Supplementary material for: A Systematic Review of the Use of Artificial Intelligence Systems for Assessing Multiple Parameters During the Ultrasound Evaluation of Fetal Growth
Source: Diagnostics (Basel). 2026 Sep 21;16(18):3063. doi: 10.3390/diagnostics16183063 (PMC13605745; doi:10.3390/diagnostics16183063)
Supplement: Supplementary file 1 [file diagnostics-16-03063-s001.zip › PROBAST AI overall conclusions.pdf]

Overall PROBAST+AI conclusions

Overall judgments for the four assessed fetal ultrasound prediction-model studies, ordered from newest to oldest.

Author and model	Development quality concerns	Development applicability concerns	Evaluation overall risk of bias	Evaluation applicability concerns
Mikołaj et al. (2025)	High concern	High concern	High	High concern
Dan et al. (2023) - DeepGA	High concern	High concern	High	High concern
Lee et al. (2023)	High concern	Low concern	High	Low concern
Fernández-Caballero et al. (2001)	High concern	High concern	High	High concern

Interpretation: High applicability concern means limited or poor applicability to the review question; low concern means good applicability. High development quality concern indicates low methodological quality. These concern ratings do not mean that an assessment item is not applicable (NA).

Mikołaj et al. (2025)

Development quality concerns — High concern. Reference-outcome timing/surrogacy and lack of recalibration after weighted training drive concern.

Development applicability concerns — High concern. Back-calculated fetal weight is not the same as observed fetal weight at the scan.

Evaluation overall risk of bias — High. Large leakage-controlled test set, but the reference outcome remains an imperfect time-dependent surrogate.

Evaluation applicability concerns — High concern. Outcome mismatch outweighs the otherwise relevant population and predictors.

Notes / unresolved issues. Reviewer disagreement concerned whether the growth-curve back-calculation is acceptable or introduces serious outcome bias.

Source supplements checked. Yes - ssupplementary material used throughout.

Dan et al. (2023) - DeepGA

Development quality concerns — High concern. Outcome incorporation and normal-only development data create major concern.

Development applicability concerns — High concern. Abnormal pregnancies are not represented in development.

Evaluation overall risk of bias — High. External evaluation is small and criterion-GA independence is uncertain.

Evaluation applicability concerns — High concern. External-case spectrum and eligibility are inadequately characterized.

Notes / unresolved issues. SegData intentionally permits images from the same patient in train and test; RegData is subject-level.

Source supplements checked. No supplement.

Lee et al. (2023)

Development quality concerns — High concern. Strong methods, but potential incorporation bias in the baseline clinical GA drives the conservative judgment.

Development applicability concerns — Low concern. Population, predictors, and GA outcome align with the review question.

Evaluation overall risk of bias — High. Held-out evaluation is strong; however, the reference GA may incorporate related fetal-biometry information.

Evaluation applicability concerns — Low concern. The two-country clinical cohort and routine inputs are relevant.

Notes / unresolved issues. Reviewer disagreement centered on whether baseline clinical GA should be considered sufficiently independent of the ultrasound predictors.

Source supplements checked. Yes; supplied Supplement 1 used.

Fernández-Caballero et al. (2001)

Development quality concerns — High concern. Single-case development with inadequate outcome definition and overfitting protection.

Development applicability concerns — High concern. The study population and outcome reference do not represent intended clinical use.

Evaluation overall risk of bias — High. Apparent performance on one case without an independent test.

Evaluation applicability concerns — High concern. Results cannot be transported to the review population.

Notes / unresolved issues. No clear train-test separation or independent fetal-weight reference.

Source supplements checked. No supplement.
