# Supplementary material for: A Systematic Review of the Use of Artificial Intelligence Systems for Assessing Multiple Parameters During the Ultrasound Evaluation of Fetal Growth
Source: Diagnostics (Basel). 2026 Sep 21;16(18):3063. doi: 10.3390/diagnostics16183063 (PMC13605745; doi:10.3390/diagnostics16183063)
Supplement: Supplementary file 1 [file diagnostics-16-03063-s001.zip › Search strategy.pdf]

Response: Thank you for highlighting this issue. To improve the reproducibility of the review, we reconstructed database-specific search strategies based on the original search concepts and predefined eligibility criteria and have provided the complete PubMed and Scopus syntax in Supplementary Table S1. The reconstructed strategies were rerun during the revision process and checked against known eligible studies to verify their sensitivity. The exact original database query history and source-specific retrieval counts were not prospectively retained; therefore, the original source-specific counts cannot be retrospectively reconstructed with certainty. This limitation has been explicitly retained in the revised manuscript.

**Database**	**Complete search strategy**	**Search rerun date**	**Limits**
PubMed	(fetal OR foetal OR prenatal) AND (ultrasound OR ultrasonography) AND ("artificial intelligence" OR "machine learning" OR "deep learning" OR "neural network" OR automated OR automatic) AND (biometry OR biometric OR measurement OR "gestational age" OR "fetal growth" OR "fetal weight")	13 Sep 2026	Publication date: 01 Jan 2000–25 Feb 2026
Scopus	TITLE-ABS-KEY ((fetal OR foetal OR prenatal) AND (ultrasound OR ultrasonography) AND ("artificial intelligence" OR "machine learning" OR "deep learning" OR "neural network" OR automated OR automatic) AND (biometry OR biometric OR measurement OR "gestational age" OR "fetal growth" OR "fetal weight"))	14 Sep 2026	Publication years: 2000–2026; eligibility restricted to records available up to 25 February 2026

Supplementary Table S1. Complete database-specific search strategies reconstructed and rerun during manuscript revision.
